# Prognostic Value of Cardiac Strain in Cognitive Impairment: A Systematic Review

**DOI:** 10.3390/medsci14020165

**Published:** 2026-03-26

**Authors:** Polyana Evangelista Lima, Anthony Rodrigues de Vasconcelos, Arthur Gabriel de Amorim Pulça, Maria Luiza de Menezes Barros, Tibério José Lopes de Alencar, Dario Celestino Sobral Filho, Paula Andreatta Maduro, Paulo Adriano Schwingel

**Affiliations:** 1 Programa de Pós-Graduação em Ciências da Saúde (PPGCS), Universidade de Pernambuco (UPE), Recife 50100-130, PE, Brazil; dario.sobral@upe.br (D.C.S.F.); paula.maduro@ebserh.gov.br (P.A.M.); 2 Laboratório de Pesquisas em Desempenho Humano (LAPEDH), Universidade de Pernambuco (UPE), Petrolina 56328-900, PE, Brazil; anthony.vasconcelos@upe.br (A.R.d.V.); arthur.pulca@discente.univasf.edu.br (A.G.d.A.P.); luiza.barros@discente.univasf.edu.br (M.L.d.M.B.); 3 Hospital de Ensino Dr. Washington Antônio de Barros da Universidade Federal do Vale do São Francisco (HU-UNIVASF), Empresa Brasileira de Serviços Hospitalares (EBSERH), Petrolina 56304-205, PE, Brazil; tiberio_alencar@hotmail.com; 4 Colegiado de Medicina, Universidade Federal do Vale do São Francisco (UNIVASF), Petrolina 56304-917, PE, Brazil; 5 Programa de Pós-Graduação em Reabilitação e Desempenho Funcional (PPGRDF), Universidade de Pernambuco (UPE), Petrolina 56328-900, PE, Brazil

**Keywords:** cognitive impairment, echocardiography, cardiac strain

## Abstract

Background/Objectives: Heart failure (HF) increases the risk of cognitive impairment (CI). Consequently, early detection and prevention of HF progression may reduce the impact of cognitive decline. The employment of novel echocardiographic imaging techniques, such as myocardial function assessment via speckle tracking strain, allows for the detection of subclinical myocardial dysfunction. The objective of this study is to identify whether speckle tracking strain has the capacity to demonstrate early cardiac alterations in patients with CI. Methods: Following a systematic review across five databases, three studies utilizing left ventricular global longitudinal strain (GLS) and seven studies utilizing left atrial global strain (LAS) were included. Results: In the assessment of myocardial strain, the sample totaled 20,614 individuals, with a mean and median age of 70 years and a predominance of females (55.3%). Left atrial global strain was the myocardial deformation marker that most frequently demonstrated an association with cognitive impairment in the listed studies. Speckle tracking GLS also demonstrated differences between groups. Only one study found no association between sensitive measures of left ventricular and left atrial function and the presence of CI. Conclusions: In conclusion, the results of this systematic review suggest that GLS and LAS are early markers of cognitive impairment.

## 1. Introduction

Heart failure (HF) increases the risk of cognitive impairment (CI), just as CI constitutes a risk factor for myocardial dysfunction and contributes to increased cardiovascular mortality [1,2]. Although both pathologies share many risk factors, are age-related, and present a progressive evolution, little is known about the association between subclinical cardiac dysfunction and cognitive impairment.

Consequently, early detection and prevention of HF progression may reduce the impact of cognitive decline. Previous studies have demonstrated an association between left ventricular ejection fraction (LVEF) and cognitive impairment [1,2,3]. However, LVEF represents an indicator of cardiac dysfunction in intermediate to advanced stages [4,5], presenting limited sensitivity, especially in the context of early detection.

The employment of novel echocardiographic imaging techniques, such as myocardial function assessment via speckle tracking strain [5,6,7], allows for the detection of subclinical myocardial dysfunction in asymptomatic individuals. This new tool aims to increase the diagnostic sensitivity of the method, particularly for incipient myocardial dysfunction. Its application is already well-established in the scenario of cardiotoxicity and the differential diagnosis of various cardiomyopathies [7,8,9].

The most widely used and reproducible parameter is global longitudinal strain (GLS), which refers to the average strain of myocardial segments in the longitudinal direction. It is capable of identifying subclinical left ventricular (LV) dysfunction and is considered a better risk predictor than LVEF [5]. In addition to GLS, studies have demonstrated the prognostic power of left atrial (LA) dysfunction via strain in predicting morbidity and mortality in the most varied clinical cardiovascular contexts [10,11].

Several articles have aimed at the early evaluation of cardiac function in individuals with CI using two-dimensional strain; the majority show worse myocardial deformation indices in individuals with CI when compared to normal controls. However, some of these studies are small and contain various confounding factors. Analyzing current evidence is necessary to synthesize existing knowledge, identify gaps, and provide robust foundations for clinical practice.

The present study aimed to identify whether two-dimensional speckle tracking echocardiography has the capacity to demonstrate early cardiac alterations in carriers of CI and its current role in the evaluation of these individuals. To this end, a systematic review was conducted on the utilization of myocardial strain techniques by echocardiography in the setting of CI.

## 2. Materials and Methods

This systematic review was conducted in accordance with the Preferred Reporting Items for Systematic Reviews and Meta-Analyses (PRISMA) 2020 statement [12]. A completed PRISMA checklist is provided as Supplementary Material Table S1. The review protocol was prospectively registered with the International Prospective Register of Systematic Reviews (PROSPERO) under the reference number CRD42023462384.

A comprehensive search was performed in MEDLINE (via PubMed), Embase, Scopus, Web of Science, and LILACS from inception to 28 October 2025, limited to studies in humans and published in English, Spanish, or Portuguese. For MEDLINE, the following search strategy was used: (‘strain’[tiab] OR ‘global longitudinal strain’[tiab] OR ‘GLS’[tiab]) AND (‘myocardium’[mh] OR ‘heart’[mh] OR myocardium[tiab] OR cardiac[tiab] OR heart[tiab]) AND (‘cognitive dysfunction’[mh] OR dementia[mh] OR ‘cognitive dysfunction’[tiab] OR ‘cognitive impairment’[tiab] OR ‘cognitive impairments’[tiab] OR ‘cognitive decline’[tiab] OR ‘cognitive defect’[tiab] OR ‘cognitive defects’[tiab] OR ‘cognitive deficit’[tiab] OR ‘cognitive disability’[tiab] OR ‘cognitive disorder’[tiab] OR ‘cognitive disorders’[tiab] OR dementia[tiab] OR ‘mental deterioration’[tiab]). Equivalent search strategies were adapted for the other databases.

The search was limited to articles on humans, in English, Spanish, and Portuguese, and was finalized on 28 October 2025. The PICOS strategy was used to define eligibility criteria: the population included adults aged over 18 years; the intervention was echocardiographic assessment via cardiac strain; there was no applicable comparator; and the outcomes of interest were cognitive dysfunction and/or dementia.

Primary studies investigating the relationship between cardiac strain parameters and cognitive impairment were included, aiming to synthesize available evidence on the association between myocardial function alterations and cognitive performance in adults. References from the databases were gathered in the Rayyan^®^ (Rayyan Systems Inc., Cambridge, MA, United States of America [USA], release 1.4.3, 2024) reference management software, and duplicates were removed.

Two researchers conducted the article’s search independently and agreed on the final selection. Initially, a broad search gathered articles related to the topic. Articles utilizing a different target population, another imaging method for measuring myocardial deformation, such as magnetic resonance imaging or tissue Doppler, the absence of GLS value description, as well as case reports, conference presentations, reviews, and editorials, were discarded through title and abstract analysis. Subsequently, the articles were accessed in full, confirming those that presented the necessary data and fit the previous criteria.

Information extracted from the articles included: author and year of publication, study design, number of sample cases, age, sex, CI assessment tools, diagnosis of hypertension and other risk factors, brand of echocardiography equipment, LVEF, and LV GLS. For studies evaluating additional echocardiographic data, left atrial volume (LAV) and left atrial strain and its three phases—reservoir deformation (LASr), conduit deformation (LAScd), and contractile deformation (LASct)—were also included. In cases of missing or unclear information, we attempted to extract data from available tables and Supplementary Materials; if unsuccessful, the variable was excluded from specific comparative analyses without imputation.

### 2.1. Data Synthesis

Given the substantial clinical and methodological heterogeneity across studies (differences in echocardiographic vendors and strain-analysis software, cognitive assessment tools, and reported cut-off values), we performed a narrative synthesis and did not pool effect estimates in a formal meta-analysis.

### 2.2. Risk of Bias Assessment

The included observational studies were appraised qualitatively with respect to selection of participants, measurement of exposure (strain parameters), measurement of cognitive outcomes, and adjustment for key confounders (age, sex, education, and cardiovascular risk factors). Given the heterogeneity of study designs and outcomes, a formal scoring system (e.g., Newcastle–Ottawa Scale) was not applied, and the risk of bias was summarized narratively in the Discussion.

## 3. Results

**Figure 1:**
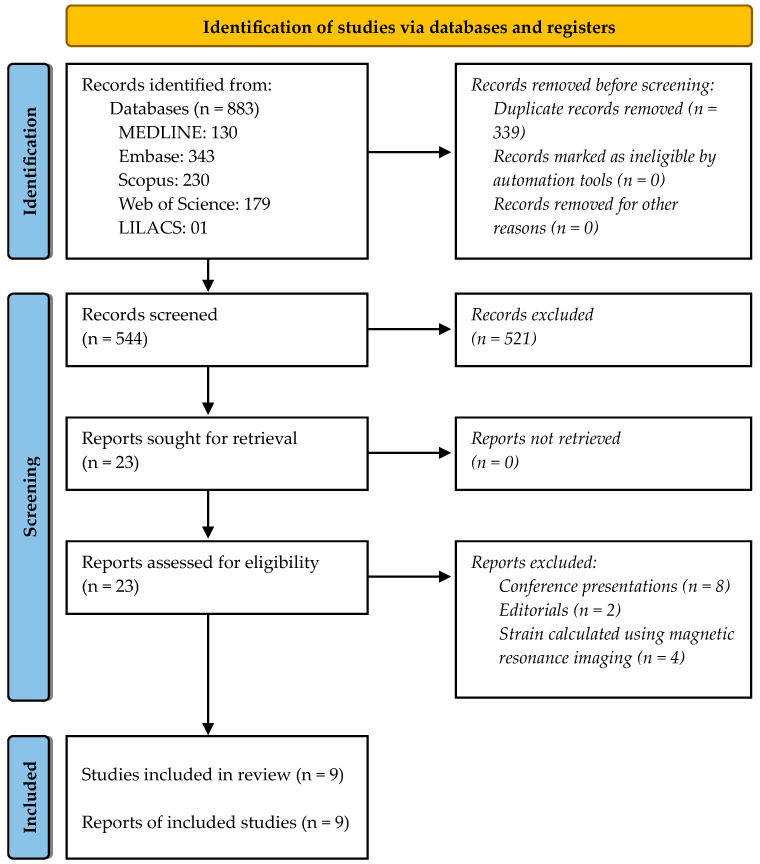
PRISMA 2020 flow diagram of study selection.

A total of 883 references were identified through electronic database searches, and 339 duplicates were discarded. Of the remaining 544 records, twenty-three full-text articles were obtained for analysis after screening titles and abstracts. Fourteen additional studies were excluded, as shown in Figure 1.

Nine articles remained for the qualitative analysis of this review, classified into two main categories: global longitudinal strain (*n* = 3) and left atrial strain (*n* = 7). Only one study evaluated both GLS and LAS [13]. All articles were published in English. Regarding the primary objective of this review, 88.8% (*n* = 8) of the analyzed studies demonstrated a significant association between impaired cardiac strain and cognitive dysfunction, regardless of the echocardiographic parameter used (GLS or LAS). Only one study reported no significant relationship between these sensitive measures of myocardial function and the presence of cognitive impairment.

Of the included studies, three were cross-sectional in nature [13,14,15], and the others performed patient follow-up (cohorts) [16,17,18,19,20,21]. In total, a population of 20,614 was included in studies utilizing the speckle tracking technique for myocardial strain assessment, with a mean and median age of 70 years. Regarding distribution by sex, there was a predominance of females, with only 44.7% of participants being male.

The most used echocardiographic equipment in these studies was the Philips iE33 (Philips Ultrasound, Inc., Bothell, WA, USA) [14,15,16,19], followed by the GE Vivid E9/E7 (GE Vingmed Ultrasound AS, Horten, Norway) [17,18,20], and only two studies utilized the Siemens ACUSON device (Siemens Medical Solutions USA, Inc., Mountain View, CA, USA) [13,21]. Regarding cognitive tests, the most used was the Mini-Mental State Examination—MMSE (PAR, Inc., Lutz, FL, USA), followed by the Montreal Cognitive Assessment—MoCA© (MoCA Test Inc., Quebec, QC, Canada) and some versions adapted for the local population [17,20].

The prevalence of cognitive decline in the included samples varied widely, ranging from 8% in a general population with metabolic syndrome to 71% in a sample of individuals followed in memory clinics in Singapore [18]. Only two studies did not report the specific number of participants with CI (Table 1); however, they presented associations between cognitive performance measures and findings of subclinical cardiac dysfunction [20,21].

Of the nine studies analyzed, the majority (*n* = 6) excluded participants with a history of stroke [14,15,16,17,20,21], and only four retained patients with atrial fibrillation in the sample [13,19,20,21]. Several risk markers associated with CI were evaluated in these studies, including sociodemographic characteristics such as age, sex, and education. The studies were homogeneous regarding age, highlighting the higher prevalence of CI with the aging process (Table 2). Regarding clinical risk factors, the high prevalence of hypertension was noted.

In addition to strain assessment via the speckle tracking technique (GLS, LASr, LAScd, LASct), two-dimensional echocardiographic characteristics were analyzed, including LVEF, LAV, LV mass, and LV diastolic function. The included articles performed statistical analyses controlling for potential confounding variables. The echocardiographic characteristics of interest for this review are presented in Table 3 and Table 4.

## 4. Discussion

The present systematic review was conducted to investigate whether myocardial strain, as assessed by two-dimensional speckle tracking echocardiography, can identify early cardiac alterations in individuals with cognitive impairment. A synthesis of evidence from nine studies, encompassing 20,614 participants, was conducted to ascertain the association between myocardial deformation parameters and cognitive dysfunction. The findings indicated a consistent association between myocardial deformation parameters, particularly left atrial strain and global longitudinal strain, and cognitive dysfunction. The results of this study suggest that cardiac strain may serve as a sensitive marker of subclinical cardiac dysfunction associated with cognitive decline. Across these included studies, 88.8% (*n* = 8) demonstrated a significant association between impaired cardiac strain (GLS or LAS) and cognitive dysfunction.

The employment of new imaging techniques for the assessment of patients with cognitive impairment has facilitated the construction of greater knowledge through the identification of incipient cardiac lesions. Left atrial global strain was the myocardial deformation marker that most frequently demonstrated an association with cognitive impairment in the listed studies [14,15,16,17,20,21].

Among its three phases, reservoir deformation (LASr) showed the strongest association, followed by conduit deformation (LAScd). A smaller number of studies utilizing contractile deformation (LASct) failed to identify differences in cognitive function over time using two-dimensional strain. Some studies established a specific LASr value for the diagnosis of subclinical atrial dysfunction, based on statistical analysis of the values obtained in the sample itself and their interquartile ranges [15,16,19].

More pronounced reductions in left atrial deformation were associated with greater severity of cognitive impairment during follow-up, independent of left atrial volume and the concomitant presence of atrial fibrillation. These findings indicate that left atrial function may play a relevant mediating role in the association between atrial fibrillation and dementia.

One study [18] proposes that left atrial strain (LAS) may contribute to the refinement of cardiovascular risk stratification, aiding in the identification of high-risk individuals for inclusion in future randomized clinical trials evaluating the indication of antithrombotic therapy aimed at reducing the risk of cerebrovascular events and secondary cognitive decline.

Regarding the echocardiographic parameter of left atrial volume, studies showed controversial results. One article found associations between the left atrial volume index and cognitive impairment [20]. However, more recent studies with longer longitudinal follow-up times pointed out that isolated left atrial enlargement, without altered atrial function, was not associated with cognitive decline [16,19].

Another available tool for subclinical myocardial function, speckle tracking GLS, also demonstrated differences between groups with cognitive impairment and healthy control groups [13,14,17]. Of the three studies that evaluated this association, two concluded that subclinical cardiac dysfunction defined by GLS is strongly correlated with cognitive decline and may provide additional information on cerebrovascular risk even when LVEF is within the normal range [14,17].

The most recent study [17], in particular, evaluates the use of GLS in hypertensive patients for the detection of mild cognitive impairment (MCI) and demonstrated an association even in this lower severity scenario, reinforcing the importance of GLS as a valuable tool for assessing preclinical cognitive and cardiac dysfunction.

When analyzing the three studies that evaluated GLS [13,14,17], it is noted that the studies used different cutoff values: <14%, <16%, and <18%. This represents a relevant source of heterogeneity among the included studies, particularly with regard to variations in echocardiographic platforms, such as the Philips iE33 [14], ACUSON [13], and GE Vivid E9 [17], and the specific software utilized for myocardial deformation analysis. The presence of variability between vendors and post-processing algorithms has the potential to influence strain measurements and cutoff values. This, in turn, can limit the direct comparability of studies. The historical absence of standardization of strain measurement cutoff points, combined with differences between equipment and analysis software, hindered direct comparison of results between studies during the initial period and limited the reproducibility of findings in the literature. However, following the publication of specific international guidelines [7], significant progress has been observed, with greater uniformity in the definition of cutoff points and better comparability of strain measurements across different platforms and studies.

The incorporation of myocardial deformation measurements into routine echocardiographic evaluation may have relevant clinical implications by enabling earlier detection of subtle cardiac dysfunction in individuals at risk of cognitive impairment [5,7]. From a public health perspective, strain imaging may improve cardiovascular risk stratification in aging populations and contribute to preventive strategies aimed at reducing the burden of both cardiovascular disease and cognitive decline [18]. The necessity of future studies is evident in order to ascertain standardized cutoff values and to evaluate whether the routine use of myocardial strain measurements can improve clinical outcomes and guide preventive interventions in this population.

In only one study [13], there was no association between sensitive measures of left ventricular and left atrial function and the presence of MCI in elderly patients (>65 years). Subclinical LV dysfunction was defined as GLS ≤ 16% and LA reservoir deformation < 24%. Subclinical LV dysfunction was found in 46% of the sample by GLS, and LA reservoir strain was abnormal in 3.6% of cases. This study found that educational attainment, systolic blood pressure, body mass index, and waist-to-hip ratio were independently associated with MCI in multivariate analysis. However, neither GLS nor LA reservoir deformation were associated with MCI. The article itself points out that this conflicting result in the literature may be due to inconsistencies in MCI diagnostic methods, as well as the small proportion of reduced atrial function (3.6%), which may have limited the study’s observations [13].

Finally, in addition to echocardiographic assessment, among the analyzed articles, the majority reinforce the role of traditional risk factors that permeate both pathologies: cardiac dysfunction and cognitive decline. Those who developed cognitive dysfunction were older, had lower education levels, and a higher prevalence of cardiovascular diseases, especially hypertension and atrial fibrillation [13,14,17,18,19].

This review has some limitations that should be considered when interpreting the findings. Notably, methodological heterogeneity was observed among the included studies, particularly regarding echocardiographic platforms, strain analysis software, and the cutoff values used for myocardial deformation parameters. Differences in study design, population characteristics, and cognitive assessment tools may also have contributed to variability in the results.

The observational design of the included studies is inherently prone to residual confounding, despite multivariable adjustment for age, education, and cardiovascular risk factors. Differences in cognitive assessment tools, strain cut-off values, and follow-up duration introduce additional sources of bias and limit causal inference. These issues should be considered when interpreting the observed associations between myocardial deformation and cognitive impairment.

Finally, future studies are needed using more standardized imaging protocols and uniform definitions.

## 5. Conclusions

In conclusion, the results of this systematic review suggest that GLS and LAS are early markers of cognitive impairment and, therefore, a valuable tool for developing preventive strategies that halt the progression of both heart disease and cognitive decline in elderly patients.

## Figures and Tables

**Table 1:** Summary of articles included in the Systematic Review, considering author of the study, participant characteristics, cognitive domain, strain measures, and their correlation with cognitive function (*n* = 9).

Study	Design	Participants	Cognitive Assessment/Domain	CI (%)	Echo/Strain/Association
Tan 2025 [18]	Prospective cohort	*N* = 251 Age: 75 ± 8 Men: 41%	MMSE; MoCA	71	Vivid E9 (GE) LVEF: 62.1 ± 5.6 LASr; LAScd; LASct Association: Positive
Ferruzzi 2025 [17]	Prospective cohort	*N* = 180 Age: 66 ± 7.2 Men: 59%	Qmci-I	22	Vivid E9 (GE) LVEF: 58 (55–60) GLS Association: Positive
Ohlrogge 2024 [21]	Prospective cohort	*N* = 7852 Age: 62 (55–69) Men: 48%	ANT; TMT-A; TMT-B; 10-WLT	—	ACUSON SC2000 (Siemens) LVEF: 59 (56–62) LASr Association: Positive
Wang 2024 [15]	Cross-sectional	*N* = 1488 Age: 76 ± 5 Men: 40%	DWRT; DSST; WFT; TICS	23	Philips iE33 LVEF: 66.3 (64.3–67.1) LASr; LAScd; LASct Association: Positive
Zhang 2023 [19]	Prospective cohort	*N* = 5461 Age: 76 (73–81) Men: 45%	LM (I/D); TMT-A; TMT-B; ANT; BNT; MMSE	14	Philips iE33 LVEF: 66.0 (62.5–69.4) LASr; LAScd; LASct Association: Positive
Wang 2022 [16]	Prospective cohort	*N* = 4096 Age: 75 ± 5 Men: 40%	DWRT; DSST; WFT; TICS	13	Philips iE33 LVEF: 66.1 (62.6–69.6) LASr Association: Positive
Gonzalez 2022 [20]	Prospective cohort	*N* = 510 Age: 65 ± 5 Men: 60%	MMSE; VFT; DST; TMT-A; TMT-B	—	Vivid E7/E9 (GE) LVEF: 65 ± 7 LASr; LAScd; LASct Association: Positive
Potter 2021 [13]	Cross-sectional	*N* = 337 Age: 70 (68–73) Men: 42%	MoCA	30	ACUSON SC2000 (Siemens) LVEF: 62 ± 6.8 GLS; LASr Association: No relation
Russo 2013 [14]	Cross-sectional	*N* = 439 Age: 69.3 ± 9.7 Men: 39%	Brain magnetic resonance imaging	12	Philips iE33 LVEF: 63.8 ± 6.4 GLS Association: Positive

Abbreviations: 10-WLT, 10-word learning test; 1st, first quartile; 3rd, third quartile; ANT, Animal Naming Test; BNT, Boston Naming Test; CI, cognitive impairment; DSST, digit symbol substitution test; DST, Digit Span Test; DWRT, delayed word recall test; GLS, global longitudinal strain, LAScd, left atrial strain (conduit deformation); LASct, left atrial strain (contractile deformation); LASr, left atrial strain (reservoir deformation); LM (I/D), Logical Memory (immediate/delayed); LVEF, left ventricular ejection fraction; MMSE, Mini-Mental State Examination; MoCA, Montreal Cognitive Assessment; Qmci-I, Italian version of the Quick Mild Cognitive Impairment screen; TICS, Telephone Interview of Cognitive Status; TMT-A, Trail Making Test Part A; TMT-B, Trail Making Test Part B; VFT, verbal fluency test; WFT, word fluency test. Data expressed as mean ± standard deviation or median (1st–3rd quartiles), and value (percentage). —, not reported.

**Table 2:** Summary of articles included in the Systematic Review, considering author of study, participant characteristics regarding risk factors (*n* = 9).

Study	*N*	Age (Years)	Men (%)	HTN (%)	HC (%)	DM (%)	Smoking (%)	Prior Stroke (%)	Prior AF (%)
Tan 2025 [18]	251	75 ± 8	41	67	73	33	20	19	EC
Ferruzzi 2025 [17]	180	65.6 ± 7.2	58.9	100	77.8	27.8	49.4	EC	EC
Wang 2024 [15]	1488	76 ± 5	40	73.8	—	30.4	4.9	EC	EC
Ohlrogge 2024 [21]	7852	62 (55–69)	48.4	63.8	—	7.6	19.6	EC	5.2
Zhang 2023 [19]	5461	76 (73–81)	42	74	52	31	—	3	22
Wang 2022 [16]	4096	75 ± 5	40	72	—	29	6	EC	EC
Gonzalez 2022 [20]	510	65 ± 5	60	83.2	100	23.7	9.4	EC	2.1
Potter 2021 [13]	337	70 (68–73)	42	87	62	32	45	6	7
Russo 2013 [14]	439	69.3 ± 9.7	39	72.7	61.5	26.2	54.2	EC	EC

Abbreviations: AF, atrial fibrillation; DM, diabetes mellitus; HC, hypercholesterolemia; HTN, hypertension. Data expressed as mean ± standard deviation or median (1st–3rd quartiles), and value (percentage). —, not reported; EC, exclusion criteria.

**Table 3:** Assessment of systolic cardiac function by two-dimensional strain using speckle tracking—GLS.

Study	Echo System	*N*	Control Group	LVEF (%)		LAV Index (mL/m^2^)		LV Hypertrophy (%)		GLS (%)	
				CI	NC	CI	NC	CI	NC	CI	NC
Ferruzzi 2025 [17]	GE Vivid E9	180	GLS > 18% (*n* = 92)	55 (54–60)	60 (55–62)	30 (26–35) **	30 (25–35)	31.8 *	20.7	MCI: 33% **	MCI: 10.9% **
Potter 2021 [13]	ACUSON SC2000	337	MCI (*n* = 101)	62 ± 5.8	62 ± 6.8	33 (29–42)	34 (28–40)	7.0	5.5	≤16	>16
Russo 2013 [14]	Philips iE33	439	SBI (*n* = 53)	63.8 ± 6.0 *	63.8 ± 6.0 *	21.9 ± 2.7 *	21.9 ± 2.7 *	—	—	<14 **	≥14

Abbreviations: CI, cognitive impairment; GLS, global longitudinal strain; LAV, left atrial volume; LV, left ventricle; LVEF, left ventricular ejection fraction; MCI, mild cognitive impairment; NC, normal cognition; SBI, silent brain infarcts. Data expressed as mean ± standard deviation, median (1st–3rd quartiles), or percentage. *, whole study population (no separate control data); **, *p* < 0.05 for difference between groups; —, not reported.

**Table 4:** Assessment of systolic cardiac function by two-dimensional strain using speckle tracking—LAS (*n* = 7).

Study	Echo System	*N*	Control Group	LVEF (%)		LAV Index (mL/m^2^)		LASr (%)		LAScd (%)		LASct (%)	
				CI	NC	CI	NC	CI	NC	CI	NC	CI	NC
Tan 2025 [18]	GE Vivid E9	251	NCI/mild CIND (*n* = 124)	62.3 ± 5.5	61.9 ± 5.8	40.1 ± 10.4	37.8 ± 8.3	29.4 ± 6.1	30.6 ± 5.7	11.6 ± 4.1 **	13.0 ± 4.0	17.8 ± 4.8	17.5 ± 4.3
Wang 2024 [15]	Philips iE33	1488	≥1 CMB (*n* = 1145)	64.3 ± 8.1	67.1 ± 4.9	28.8 ± 9.7	23.4 ± 5.8	27.4 *	37.6	10.7 *	18.0	14.4	21.1
Ohlrogge 2024 [21]	ACUSON SC2000	7852	HCHS Cohort (*n* = 7852)	58.5 (55.5– 61.8) #	58.5 (55.5– 61.8) #	25.2 (20.4– 30.8) #	25.2 (20.4– 30.8) #	38.0 (29.9– 48.3) ¶	38.0 (29.9– 48.3) #	—	—	—	—
Zhang 2023 [19]	Philips iE33	5461	No dementia with AF (*n* = 1205)	66.0 (62.5– 69.4)	64.3 (59.6– 68.1)	31.7 (25.6– 38.9)	39.0 (30.2– 48.2)	33.2 ± 7.4 †	24.6 ± 10.1	15.0 ± 5.7 ‡	12.4 ± 5.2	18.2 ± 5.5 §	12.5 ± 7.5
Gonzalez 2022 [20]	GE Vivid E7/ Vivid E9	510	MetS adults (*n* = 510)	65.4 ± 7.0 #	65.4 ± 7.0 #	23.2 ± 7.6 #	23.2 ± 7.6 #	27.2 ± 6.8 *	27.2 ± 6.8 #	11.9 ± 4.4 *	11.9 ± 4.4 #	15.3 ± 5.2 *	15.3 ± 5.2 #
Wang 2022 [16]	Philips iE33	4096	No dementia (*n* = 3565)	65.9 (61.5– 69.0)	66.1 (62.6– 69.6)	33.6 (26.7– 42.3)	32.0 (25.9– 39.4)	30.1 ± 8.1 *	33.2 ± 7.5	13.1 ± 5.4 *	15.1 ± 5.6	17.0 ± 5.8 *	18.1 ± 5.5
Potter 2021 [13]	ACUSON SC2000	337	MCI (*n* = 101)	62 ± 5.8	62 ± 6.8	33 (29– 42)	34 (28– 40)	<24%	≥24%	—	—	—	—

Abbreviations: AF, atrial fibrillation; CI, cognitive impairment; CIND, cognitive impairment without dementia; CMB, cerebral microbleed; HCHS, Hamburg City Health Study; LAScd, left atrial strain (conduit deformation); LASct, left atrial strain (contractile deformation); LASr, left atrial strain (reservoir deformation); LAV, left atrial volume; LVEF, left ventricular ejection fraction; MCI, mild cognitive impairment; MetS, metabolic syndrome; NCI, no cognitive impairment; NC, normal cognition. Data expressed as mean ± standard deviation, median (1st–3rd quartiles), or percentage. *, *p* < 0.05; **, *p* = 0.005; †, *p* = 0.002 (adjusted *p* = 0.23); ‡, *p* = 0.002 (adjusted *p* = 0.005); §, *p* = 0.002 (adjusted *p* = 0.12); #, whole cohort (no separate control); ¶ *p* = 0.001 in TMT-A and TMT-B; —, not reported.

## Data Availability

The original contributions presented in this study are included in the article. Further inquiries can be directed at the corresponding author.

## Supplementary Materials

The following supporting information can be downloaded at: https://www.mdpi.com/article/10.3390/medsci14020165/s1, Table S1: PRISMA 2020 Checklist [22].

## Abbreviations

The following abbreviations are used in this manuscript:

10-WLT	10-word learning test
1st	First quartile
3rd	Third quartile
AF	Atrial fibrillation
ANT	Animal Naming Test
BNT	Boston Naming Test
CI	Cognitive impairment
CIND	Cognitive impairment without dementia
DSST	Digit symbol substitution test
DST	Digit Span Test
DWRT	Delayed word recall test
GLS	Global longitudinal strain
HF	Heart failure
LA	Left atrium (left atrial)
LAS	Left atrial strain
LAScd	Left atrial strain (conduit deformation)
LASct	Left atrial strain (contractile deformation)
LASr	Left atrial strain (reservoir deformation)
LAV	Left atrial volume
LM (I/D)	Logical memory (immediate/delayed)
LV	Left ventricle (left ventricular)
LVEF	Left ventricular ejection fraction
M	Mean
MCI	Mild cognitive impairment
MMSE	Mini-Mental State Examination
MoCA	Montreal Cognitive Assessment
PRISMA	Preferred Reporting Items for Systematic Reviews and Meta-Analyses
PROSPERO	International Prospective Register of Systematic Reviews
Qmci-I	Italian version of the Quick Mild Cognitive Impairment screen
SBI	Silent brain infarcts
SD	Standard deviation
TICS	Telephone Interview of Cognitive Status
TMT-A	Trail Making Test Part A
TMT-B	Trail Making Test Part B
UPE	University of Pernambuco
VFT	Verbal fluency test
WFT	Word fluency test
