# Supplementary material for: Prognostic Value of Cardiac Strain in Cognitive Impairment: A Systematic Review
Source: Med Sci (Basel). 2026 Mar 26;14(2):165. doi: 10.3390/medsci14020165 (PMC13108025; doi:10.3390/medsci14020165)
Supplement: Supplementary file 1 [file medsci-14-00165-s001.zip › medsci-4182069-supplementary.pdf]

PRISMA 2020 Checklist

Section and Topic	Item #	Checklist item	Location where item is reported
**TITLE**			
Title	1	Identify the report as a systematic review.	The report is identified as a systematic review in the title.
**ABSTRACT**			
Abstract	2	See the PRISMA 2020 for Abstracts checklist.	A structured abstract (Background/Objectives, Methods, Results, Conclusions) summarises the review in accordance with PRISMA 2020 for Abstracts.
**INTRODUCTION**			
Rationale	3	Describe the rationale for the review in the context of existing knowledge.	The rationale for the review in the context of existing knowledge is described in the Introduction.
Objectives	4	Provide an explicit statement of the objective(s) or question(s) the review addresses.	The explicit objective of the review is stated in the final paragraph of the Introduction.
**METHODS**			
Eligibility criteria	5	Specify the inclusion and exclusion criteria for the review and how studies were grouped for the syntheses.	In the Methods section, the inclusion and exclusion criteria and the PICOS framework are specified, including population (adults ≥18 years), echocardiographic strain assessment, cognitive impairment/dementia outcomes, language, and study design.
Information sources	6	Specify all databases, registers, websites, organisations, reference lists and other sources searched or consulted to identify studies. Specify the date when each source was last searched or consulted.	The Methods section lists all databases searched (MEDLINE/PubMed, Embase, Scopus, Web of Science, LILACS) and reports that the search was conducted up to 28 October 2025.
Search strategy	7	Present the full search strategies for all databases, registers and websites, including any filters and limits used.	The full MEDLINE search strategy is presented in the Methods section, and equivalent strategies are described as adapted for the other databases.
Selection process	8	Specify the methods used to decide whether a study met the inclusion criteria of the review, including how many reviewers screened each record and each report retrieved, whether they worked independently, and if applicable, details of automation tools used in the process.	The Methods section describes how two reviewers independently screened titles/abstracts and full texts using Rayyan and applied the eligibility criteria to decide which studies to include.
Data collection process	9	Specify the methods used to collect data from reports, including how many reviewers collected data from each report, whether they worked independently, any processes for obtaining or confirming data from study investigators, and if applicable, details of automation tools used in the process.	The Methods section explains that data were extracted independently by two reviewers using predefined variables and that disagreements were resolved by consensus.
Data items	10a	List and define all outcomes for which data were sought. Specify whether all results that were compatible with each outcome domain in each study were sought (e.g. for all measures, time points, analyses), and if not, the methods used to decide which results to collect.	The Methods section lists and defines the primary outcomes related to cognitive impairment and dementia, including the cognitive tests and diagnostic criteria used in the included studies.
	10b	List and define all other variables for which data were sought (e.g. participant and intervention characteristics, funding sources). Describe any assumptions made about any missing or unclear information.	The Methods section lists and defines other variables extracted (age, sex, cardiovascular risk factors, echocardiographic parameters, equipment type, left ventricular ejection fraction, GLS, LAS and related indices) and describes how missing or unclear data were handled.
Study risk of bias assessment	11	Specify the methods used to assess risk of bias in the included studies, including details of the tool(s) used, how many reviewers assessed each study and whether they worked independently, and if applicable, details of automation tools used in the process.	The Methods subsection 'Risk of Bias Assessment' states that the observational studies were appraised qualitatively regarding selection, exposure measurement, outcome assessment, and adjustment for key confounders, without applying a formal scoring tool.

Section and Topic	Item #	Checklist item	Location where item is reported
Effect measures	12	Specify for each outcome the effect measure(s) (e.g. risk ratio, mean difference) used in the synthesis or presentation of results.	No single effect measure was used for pooling; instead, study-level statistics (e.g., p-values, correlation coefficients, hazard ratios or odds ratios) are reported in the text and in Tables 1, 3 and 4. No meta-analysis was performed.
Synthesis methods	13a	Describe the processes used to decide which studies were eligible for each synthesis (e.g. tabulating the study intervention characteristics and comparing against the planned groups for each synthesis (item #5)).	The 'Data Synthesis' subsection describes how studies were grouped qualitatively according to the main echocardiographic strain parameter (GLS versus LAS) for narrative synthesis.
	13b	Describe any methods required to prepare the data for presentation or synthesis, such as handling of missing summary statistics, or data conversions.	No special methods were required to prepare data for synthesis; the review reports summary statistics as presented in the original studies, without data conversion or imputation.
	13c	Describe any methods used to tabulate or visually display results of individual studies and syntheses.	The Methods and Results sections describe and present the characteristics and results of individual studies in structured tables (Tables 1–4) and a PRISMA 2020 flow diagram (Figure 1).
	13d	Describe any methods used to synthesize results and provide a rationale for the choice(s). If meta-analysis was performed, describe the model(s), method(s) to identify the presence and extent of statistical heterogeneity, and software package(s) used.	The 'Data Synthesis' subsection explains that, due to substantial clinical and methodological heterogeneity, a narrative synthesis was undertaken, and no meta-analysis was conducted, with justification provided.
	13e	Describe any methods used to explore possible causes of heterogeneity among study results (e.g. subgroup analysis, meta-regression).	The Discussion section narratively explores sources of heterogeneity, including differences in echocardiographic platforms, strain-analysis software, cut-off values, study designs, populations and cognitive assessment tools.
	13f	Describe any sensitivity analyses conducted to assess robustness of the synthesized results.	No sensitivity analyses were conducted because no quantitative synthesis or meta-analysis was performed.
Reporting bias assessment	14	Describe any methods used to assess risk of bias due to missing results in a synthesis (arising from reporting biases).	No formal assessment of reporting bias (e.g., publication bias) was carried out, as no statistical synthesis of effect estimates was undertaken.
Certainty assessment	15	Describe any methods used to assess certainty (or confidence) in the body of evidence for an outcome.	No formal assessment of certainty or confidence in the body of evidence (such as GRADE) was performed.
**RESULTS**			
Study selection	16a	Describe the results of the search and selection process, from the number of records identified in the search to the number of studies included in the review, ideally using a flow diagram.	The Results section reports the numbers of records identified, screened, excluded and included, and presents a PRISMA 2020 flow diagram (Figure 1).
	16b	Cite studies that might appear to meet the inclusion criteria, but which were excluded, and explain why they were excluded.	The Results section and the PRISMA flow diagram indicate the number of full-text articles excluded and the main reasons for exclusion, although individual excluded studies are not listed.
Study characteristics	17	Cite each included study and present its characteristics.	The characteristics of each included study (design, setting, participants, risk factors, cognitive assessments, echocardiographic parameters and sample sizes) are presented in the Results section and in Tables 1–4.
Risk of bias in studies	18	Present assessments of risk of bias for each included study.	The Discussion section summarises concerns about risk of bias, including the observational design of the studies, residual confounding and methodological variability; overall risk-of-bias judgements are not presented for each individual study.

PRISMA

PRISMA 2020 Checklist

Section and Topic	Item #	Checklist item	Location where item is reported
Results of individual studies	19	For all outcomes, present, for each study: (a) summary statistics for each group (where appropriate) and (b) an effect estimate and its precision (e.g. confidence/credible interval), ideally using structured tables or plots.	For each study, summary statistics and the presence or absence of significant associations between strain measures and cognitive outcomes are presented in Tables 1, 3 and 4 and described narratively in the Results section.
Results of syntheses	20a	For each synthesis, briefly summarise the characteristics and risk of bias among contributing studies.	The Results section summarises the overall evidence from the nine included studies (20,614 participants, 8 of 9 with significant associations), and the Discussion addresses methodological heterogeneity and risk-of-bias issues across studies.
	20b	Present results of all statistical syntheses conducted. If meta-analysis was done, present for each the summary estimate and its precision (e.g. confidence/credible interval) and measures of statistical heterogeneity. If comparing groups, describe the direction of the effect.	No statistical syntheses or meta-analyses were performed; therefore, no pooled effect estimates or heterogeneity statistics are reported.
	20c	Present results of all investigations of possible causes of heterogeneity among study results.	No formal statistical investigation of heterogeneity (such as subgroup analysis or meta-regression) was performed; only narrative exploration is provided in the Discussion.
	20d	Present results of all sensitivity analyses conducted to assess the robustness of the synthesized results.	No sensitivity analyses were conducted.
Reporting biases	21	Present assessments of risk of bias due to missing results (arising from reporting biases) for each synthesis assessed.	Risk of bias due to missing results (reporting biases) was not formally assessed because no quantitative synthesis of effect estimates was undertaken.
Certainty of evidence	22	Present assessments of certainty (or confidence) in the body of evidence for each outcome assessed.	The certainty or confidence in the body of evidence was not formally graded.
**DISCUSSION**			
Discussion	23a	Provide a general interpretation of the results in the context of other evidence.	The Discussion provides an overall interpretation of the findings considering current evidence on myocardial strain and cognitive impairment.
	23b	Discuss any limitations of the evidence included in the review.	The Discussion outlines limitations of the included evidence, such as observational designs, residual confounding, heterogeneity in imaging platforms, strain cut-offs and cognitive assessments.
	23c	Discuss any limitations of the review processes used.	The Discussion briefly acknowledges limitations of the review process, including language restriction to English, Spanish and Portuguese and the inability to perform a meta-analysis due to heterogeneity.
	23d	Discuss implications of the results for practice, policy, and future research.	The Discussion considers the implications of the findings for clinical practice and future research, including the potential role of strain imaging in risk stratification and the need for standardized protocols and prospective studies.
**OTHER INFORMATION**			
Registration and protocol	24a	Provide registration information for the review, including register name and registration number, or state that the review was not registered.	The Methods section reports that the review was prospectively registered in PROSPERO with registration number CRD42023462384.
	24b	Indicate where the review protocol can be accessed, or state that a protocol was not prepared.	The Methods section notes the PROSPERO registration number, and the full protocol can be accessed via the PROSPERO record.

PRISMA

PRISMA 2020 Checklist

Section and Topic	Item #	Checklist item	Location where item is reported
	24c	Describe and explain any amendments to information provided at registration or in the protocol.	No amendments to the registered protocol are reported.
Support	25	Describe sources of financial or non-financial support for the review, and the role of the funders or sponsors in the review.	The Funding section describes all financial and institutional support and clarifies the role of funders (CAPES, CNPq and institutional support) in the review.
Competing interests	26	Declare any competing interests of review authors.	The Conflicts of Interest section declares that the authors have no competing interests.
Availability of data, code and other materials	27	Report which of the following are publicly available and where they can be found: template data collection forms; data extracted from included studies; data used for all analyses; analytic code; any other materials used in the review.	The Data Availability Statement indicates that all data supporting the findings are contained within the article; additional data extraction forms and analytic code are not publicly archived.

From: Page MJ, McKenzie JE, Bossuyt PM, Boutron I, Hoffmann TC, Mulrow CD, et al. The PRISMA 2020 statement: an updated guideline for reporting systematic reviews. BMJ 2021;372:n71. doi: 10.1136/bmj.n71. This work is licensed under CC BY 4.0. To view a copy of this license, visit https://creativecommons.org/licenses/by/4.0/
